# Efficacy and Safety of Intra-Articular Therapy with Cross-Linked Hyaluronic Acid in Patients with Knee Osteoarthritis

**DOI:** 10.3390/ph18030302

**Published:** 2025-02-22

**Authors:** Vincenzo Rania, Gianmarco Marcianò, Cristina Vocca, Caterina Palleria, Luigi Bianco, Maria Cristina Caroleo, Luca Gallelli

**Affiliations:** 1 Operative Unit of Pharmacology and Pharmacovigilance, “Renato Dulbecco” University Hospital, 88100 Catanzaro, Italy; raniavincenzo1@gmail.com (V.R.); gianmarco.marciano3@gmail.com (G.M.); cristina_vocca@live.it (C.V.); palleria@unicz.it (C.P.); luigibianco1969@gmail.com (L.B.); mariacristina.caroleo@unicz.it (M.C.C.); 2 Research Center FAS@UMG, Department of Health Science, University Magna Graecia, 88100 Catanzaro, Italy

**Keywords:** hyaluronic acid, single injection, cross-linked, knee osteoarthritis

## Abstract

**Background/Objectives**: Knee osteoarthritis (OA) is a degenerative chronic disease characterized by a reduction in articular cartilage, as well as pain and functional limitations. We evaluated both the efficacy and safety of cross-linked high-molecular-weight hyaluronic acid in patients with knee OA. **Methods:** In this observational prospective single-arm study, a cross-linked high-molecular-weight hyaluronic acid (DIART ONE 90 mg in 3 mL) was administered in single injections to 50 patients aged 18–65 years, with a follow-up at 3, 6, and 12 months. Several scores were evaluated, including the Knee Injury and Osteoarthritis Outcome Score as the primary outcome measure and the Visual Analogue Scale, Timed Up and Go Test, Six-Minute Walking Test, General Health Assessment with 36-Item Short Form Health Survey, Zung’s Self-Rating Anxiety Scale, and Zung’s Self-Rating Depression Scale as secondary outcome measures. Both physicians and patients knew the kind of treatment they received. **Results**: During the follow-ups, we observed a statistically significant improvement in clinical scores at 3 and 6 months, with a decrease in clinical benefit at 12 months. Functional and psychological benefits were significant at 3, 6, and 12 months. No side effects were described except pain associated with the site of injection. **Conclusions:** In conclusion, we documented that cross-linked high-molecular-weight hyaluronic acid (DIART ONE 90 mg in 3 mL) represents an effective option in the management of mild–moderate osteoarthritis.

## 1. Introduction

According to the Global burden of Disease study, the age-standardized point prevalence and annual incidence rates of osteoarthritis (OA) in 2017 were 3754.2 (95% UI 3389.4 to 4187.6) and 181.2 (95% UI 162.6 to 202.4) per 100,000, an increase of 9.3% (95% UI 8% to 10.7%) and 8.2% (95% UI 7.1% to 9.4%) from 1990. The global prevalence estimate was higher in women and increased with age, peaking for the >95 age group for both women and men in 2017 [1].

The number of people with symptomatic knee OA is likely to increase due to aging and obesity [2,3,4]. Although the disease pathophysiology is not completely understood and is still under investigation, it is accepted that knee OA has a multifactorial origin inducing articular cartilage damage, bone osteophyte formation, sclerosis of the subchondral bone, and, in advanced cases, subchondral cyst formation [5]. Currently, research is focused on novel therapeutic strategies to repair cartilage in order to restore function and reduce pain. These techniques need to take into account the frictional and mechanical properties of cartilage. Despite research efforts, total joint replacement (TJR) is still the only treatment available for OA’s advanced stages [6].

The most common symptom of knee OA is continuous or intermittent pain usually related to the severity of articular damage, with swelling, locking, and instability as common related symptoms [7,8].

Diagnosis is based on clinical examination and imaging studies, e.g., ultrasonography, radiography, computed tomography and magnetic resonance imaging [9].

According to the international recommendations, the management of knee OA is chosen considering the patient’s characteristics, the degree of severity of the OA itself, and the pain related to it, and it requires a combination of non-pharmacological and pharmacological treatments [10,11,12,13,14]. Several behavioral factors may determine OA onset and therapeutic response, e.g., job, sports, musculoskeletal injuries, obesity, and gender [6]. Sex and gender are associated with significant differences in OA prevalence and management [6]. In fact, women generally use more health care, and are more frequently affected by OA, pain and inflammation. Furthermore, compared to men, women have physical limitations, decreased cartilage volume, and smaller joint parameters and dimensions in a higher percentage of cases [15]. Finally, anatomic, biomechanical and hormonal factors that differ between women and men (i.e., limb alignment, estrogen levels, lower muscle strength, alcohol consumption, and atherosclerosis) might predispose women to knee OA [16,17].

Concerning obesity, this is a complex clinical condition that may lead to OA both in weight-bearing and in non-weight bearing joints. This pathology doubles the risk of OA in comparison to healthy people. It is important to consider the role of obesity not only in increasing joint efforts, but also in producing several inflammatory mediators. According to these mechanisms, degradation and synthesis to rebuild the damaged tissue become a constant process in the joints. Despite the important role of mechanical stress, inflammatory cells were found to be present in synovia membranes. The production of adipokines due to increased adipose tissue has a role in regulating several functions, including inflammation. Inflammatory response has been found to be different in lean and obese people. For example, the expression of T lymphocytes is different: that of Th1, Th17, T helper, and B cells are lower in comparison with that of Treg cells and NK cells in lean people [18].

In obesity, the opposite happens. Macrophage expression is also different since the concentrations of M2 cells are higher in lean people, whereas those of M1 cells are higher in obese people. Moreover, obese people have higher levels of neutrophils and a lower numbers of eosinophils [18].

In a retrospective study, it was documented that patients living in underprivileged areas have a higher incidence of OA than those in more privileged areas [19]. Workers in construction, agriculture, firefighting, mining, fisheries, and forestry have an increased risk of OA. Repetitive and heavy activity seems to have the potential to sustain chronic damage, generating long-term injury. Other factors include vibrations, kneeling, bending, regular stair climbing, jumping, weight lifting, standing on a rigid surface and crawling [20,21]. According to several studies, the risk of OA is increased fivefold when risk factors like weightlifting and squatting/climbing stairs are combined. Age and occupational damage are strongly correlated since work appears to have a major role in the start of OA, primarily in people over 50. In spite of the elevated risk of obesity, sedentary labor was linked to a lower risk of OA than intensive work, and after a number of years, the overall risk of OA increased only marginally with sedentary jobs [20,22].

Lastly, given the potential for musculoskeletal damage to more knee ligaments, muscles, or tendons, some sports, such as basketball, football, volleyball, weightlifting, professional long-distance running, and wrestling, are linked to an increased risk of osteoarthritis. Meniscal or ligament injury leads to joint instability, with an increased risk of osteoarthritis [23]. Tran et al. [24], reviewing 46 studies, reported an increased risk of OA in patients performing sports (RR 1.37; 95% CI 1.14 to 1.6). However, no differences were found in terms of risk factors between non-professional and elite athletes. In this review, the authors documented a relationship with the intensity of physical activity and with meniscectomies or anterior ligament tears, since the evidence is not compelling.

Even if systemic drugs can be used to reduce pain, their use could be associated with noteworthy side effects, especially when used in a chronic setting [25].

Therefore, topical intra-articular injections represent an important option depending on both joint characteristics and practitioner skills. Among the injective therapeutic options, corticosteroids, hyaluronic acid (HA), and platelet-rich plasma are the most common [10,26].

Hyaluronic acid is a polysaccharide with important actions in the synovial fluid and cartilage, including lubrication, protection of cartilage, and shock absorption of the joint [27]. Formulations of HA may vary in their weight (low or high), presence of cross-linking, number of injections and production source [28]. Its action is not limited to mechanical properties, but it can provide a great variety of anti-inflammatory effects through its binding to CD44 type I transmembrane glycoprotein [28,29,30].

Hyaluronic acid intra-articular injection is a cornerstone of knee OA management since it provides symptom relief and delays time to prosthesis implantation, with low risks of adverse drug reactions [31] that are commonly represented by local pain or infections increasing with repetitive administrations [27,32].

Therefore, the administration of a single injection of a cross-linked high-molecular-weight (HMW) HA could be useful in improving clinical symptoms in patients with OA, reducing the ADRs. Cross-linking is a chemical process that modifies the natural straight-chain structure of the molecule, producing an entangled HA molecule. This structural change may substantially increase the effectiveness of HA formulations [28].

Ferkel et al. [28] showed that HMW-HA has a greater probability of modulating this pathway than LMW and greater clinical efficacy especially after 3 and 6 months. Nevertheless, no comparison between single injections and triple-injection HMW-HA has been made. The same authors observed that avian-derived and cross-linked HA were associated with a higher inflammatory reaction compared with non-avian and non-cross-linked HA. Cross-linking results in a different interaction with CD44 and a gel-like state that is different from that of human HA. Despite this, cross-linking may confer an advantage due to its rheological properties and increased residence in the joint.

In agreement, Rutjes and colleagues [33] observed that cross-linking HMWHA determines better clinical outcomes. Nevertheless, it has not been proven if this effect is related to cross-linking or simply to HMW and studies comparing similar-weighted (cross-linked vs. non-cross-linked) HA are lacking.

In this study, we evaluated the efficacy and the safety of the intra-articular injection of a cross-linked high-molecular-weight hyaluronic acid in improving the symptoms and function of patients with OA.

## 2. Results

### 2.1. Patients

Seventy-four patients with knee OA were enrolled between 1 March 2023 and 31 May 2023. Twenty-four patients (32.4%) were excluded because they did not meet the study inclusion criteria. In total, 50 patients, 14 females (28%) and 36 males (72%), underwent the treatment and signed the informed consent form (Table 1).

### 2.2. Effects on Pain

At T0, the mean value of VAS was 6.90 ± 1.23, and that of KOOS was 44.90 ± 14.10; while the walking test was positive after 3 min. The result from the Timed Up and Go Test at this time was 5.0 ± 2.1 s. At T1, we documented a significant improvement in the results for the VAS, KOOS, and Timed Up and Go Test (Table 2). At T2, we recorded a further improvement in clinical symptoms that was maintained for the following months, during which we recorded a significant improvement in walking ability and in results of the Timed Up and Go Test. At T3, we also observed a worsening of the KOOS and VAS values compared with those at T2 (Table 3). A sub-analysis of pain effects highlighted no difference between males and females in pain improvement (*p* > 0.05).

### 2.3. Effects on Quality of Life

At T1, the SF-36 questionnaire score revealed a time-related significant improvement in the quality of life (*p* < 0.01) (Table 4). Moreover, we also recorded a statistically significant (*p* < 0.01) improvement in the SF-36 score during the follow-ups (T1–T2) (Table 5), with a worsening of outcomes at 1 year (Table 6).

### 2.4. Effect on Mood Symptoms

Using both the Zung SDS (depression) and Zung SAS (anxiety) scales, we observed a statistically significant improvement in mood disorders (*p* < 0.01) (Table 7). Moreover, statistical evaluation of the data recorded in T3 vs. T2 revealed a statistically significant worsening of symptoms (*p* < 0.01) since the patients reported an improvement in their symptoms compared with those at admission.

### 2.5. Safety

We did not document any adverse drug reactions during the study, except for injection site pain in three patients, which did not impair study adherence. Moreover, up until December 2024, about 6 months after the later follow-up, we did not record the development of any adverse drug reactions in the enrolled patients.

## 3. Discussion

In the present study, we documented that in knee OA patients, the injection of cross-linked high-molecular-weight HA (90 mg in 3 mL) induced an improvement in clinical symptoms without the development of adverse drug reactions. We also evaluated the effects of this formulation, looking for whether there was an association between corticosteroid and anesthetic use.

Knee osteoarthritis is a common musculoskeletal disorder causing significant loss of joint function, with pain, inflammation, and dysfunction due to both progressive articular cartilage degeneration and synovitis [34].

Even if several drugs can be used to reduce pain and/or inflammation (e.g., acetaminophen or NSAIDs), topical treatment is suggested to reduce the development of adverse drug reactions, particularly in patients with comorbidity and polytherapy [12].

In our study, we enrolled participants with chronic knee OA who did not experience a clinical improvement in symptoms with common systemic or local compounds (we also considered those at T0 as the control group). Patients came to our pain clinic room with a diagnosis of OA in their history, as determined via clinical examination and radiography. According to OA recommendations [35,36,37] and our hospital’s standard clinical practice, patients did not receive magnetic resonance imaging (MRI) or vibroarthrography. 

In these patients intra-articular treatment with cross-linked high-molecular-weight HA induced a significant decrease in pain and a significant increase in the quality of life with an improvement in the KOOS score and in other clinical parameters. These effects are related to the mechanism of actions of both compounds, which are able to reduce inflammation and pain. In particular, HA treatment has anti-inflammatory properties in addition to mechanical activity in articulations [38]. Although HA interacts with other structures such as lymphatic vessel endothelium receptor 1, Intercellular Adhesion Molecule, receptor for hyaluronan-mediated motility, and tool-like receptors, the primary receptor for HA is CD44. The cascade that results from the binding of HA and CD44 has subchondral, chondroprotective, anti-inflammatory, and proteoglycan production effects, with a decrease in the breakdown of joint cartilage and a decrease in interleukin-1β and metalloproteinases [38].

The first use of intra-articular cortisone dates back to 1951 [39], and some studies have documented its efficacy in a short time in patients with OA due to its anti-inflammatory and immunosuppressive effects [40].

In the present study, intra-articular treatment with triamcinolone acetonide, a long-half-life corticosteroid (half-life 3.2–6.4 days), induced a significant improvement in clinical symptoms that decreased three months later. This effect was lower with respect to those observed with the use of the cross-linked high-molecular-weight HA formulation. This is could be related to the type of HA used; in fact, the cross-linked high-molecular-weight HA formulation has anti-inflammatory, chondroprotective and mechanical effects, with better clinical outcomes [33]. An interesting study by Perruchet et al. [41] evaluated the factors conditioning responses to a single injection of extended release (HA HANOX-M-XL) in 51 patients. The primary outcome was the self-measurement of the duration of effectiveness (DE). They found that, like gender (longer durations in men, *p* = 0.02) and older age (*p* = 0.04), K-L grade was a significant factor in determining DE (*p* = 0.007). For almost a year, even patients with severe OA demonstrated improvement. Patients with K-L III and IV did not have different outcomes, even though those with the latter typically require surgery. This was most likely due to the inclusion of participants who had previously received cycles of HA injections and those who did not exhibit severe symptoms. Additionally, contrary to previous research, obesity and body mass index were not linked to a worse outcome. In our study, we analyzed sex differences in pain outcomes, but we did not show significant differences between males and females. This was mainly because of the reduced number of women and the relatively low mean age (49 years). In fact, knee osteoarthritis is estimated to be more severe and frequent in women after menopause, due to the loss of the protective effect of estrogens [42,43,44]. Despite this, other authors failed to find a correlation between menopause and OA severity [45]. Concerning OA severity, we did not perform any statistical analysis due to the low number of patients in class II/III and III. Similarly, the absence of elderly or obese people in our group did not allow us to perform true stratification or to obtain useful results from these variables.

In our study, we excluded patients with a Body Mass Index lower than 29 kg/m^2^, and this represents the novelty of our study because some authors have reported that factors like obesity may reduce HA efficacy [46]. Moreover, in our study, we did not report the development of serious adverse drug reactions even though the long-time use of corticosteroids may induce the development of side effects [40].

This could have been due to two factors: (1) the short duration of treatment with corticosteroids; (2) the use of a single injection of high-molecular-weight cross-linking HA, as well as the fact that this formulation was associated with high safety; the advantage of these choices became clear in patients managed with anticoagulants, busy subjects or those not able to tolerate three injections.

Concerning the safety of hyaluronic acid, we observed that the most common side effect was pain in the site of injection. Rutjes et al. [33] failed to show differences in the side effects between HA-treated people and controls. A possible inflammatory side effect related to HA injection is pseudo-sepsis, characterized by inflammation of the joint, effusion and pain 24–72 h after injection. Pseudosepis is characterized by the impossibility of detecting a pathogen or calcium pyrophosphate crystals in the synovial fluid. This adverse event usually requires NSAIDs or arthrocentesis/steroid injections to be managed [47]. Overall, HA (available on the market in different shapes from several years) is generally considered safe by the scientific community, and its role in the cartilage is estimated to be protective and regenerative, considering the relative rarity of this inflammatory reaction [48]. Despite all these considerations and the improbability of severe side effects, long-term monitoring and pharmacovigilance are required, especially for new formulations on the market.

The effectiveness and safety of HA in comparison with those of other intra-articular treatments for knee OA, such as platelet-rich plasma (PRP) and corticosteroids, should also be considered. Qiao and colleagues [26] documented that, in OA patients, PRP and PRP + HA were the most effective options in improving pain and function at 3, 6 and 12 months, without the development of adverse events. The same results were reported in another meta-analysis [49].

We must consider certain practical considerations other than the effectiveness of PRP in tissue regeneration and the significant impact of corticosteroids in lowering inflammation. Corticosteroids only work for 7–14 days, and using them can cause a number of negative side effects, such as weight gain, diabetes, high blood pressure, and osteoporosis [40,50]. Additionally, blood must be drawn for PRP, and not all patients will tolerate it well. Additional potential problems or contraindications for this technique include a history of cancer (growth factors in PRP may exacerbate patients’ conditions), a change in blood count (insufficient platelets or the presence of significant hematological diseases), or antiplatelet therapy (potential reductions in PRP’s efficacy) [51,52]. These studies suggest that HA has been shown to be effective in every study, it is one of the most significant treatment choices. Additional information about cost-effectiveness has been provided [53,54]. PRP and HA are both cost-effective (cost per quality-adjusted life-year (QALY): PRP, USD 8635.23/QALY; HA, USD 5331.75/QALY; total cost per QALY, < USD 50,000) [53]. However, after a year of numerous injections, PRP is more cost-effective (difference: USD 1433.67) than HA (0.69 QALYs vs. 0.58 QALYs, *p* = 0.0062), with an incremental cost-effectiveness ratio of USD 12,628.15/QALY vs. HA.

Corticosteroid treatment is less expensive in terms of costs, but total expenses may increase due to both side effects and the short duration of efficacy [26,55].

In our study, the long-duration follow-up (December 2024) revealed that high-molecular-weight cross-linking HA is a safe compound, since we documented the loss of efficacy 12 months after the administration. Since we were not able to demonstrate the biochemical modification related to this impairment, we can deduce that the time-related degradation of the cross-linking HA formulation induced the loss of biomechanical activity. However, this formulation improves the clinical symptoms for a long time, which could reduce health costs.

Even though we did not evaluate the cost of the treatment, during the study, the enrolled patients did not use other medications for pain management. Ranawat et al. [56] showed that a hyaluronic acid formulation with a single injection is cost-effective because it reduces the need for the use of co-medications (e.g., opioids, NSAIDs and corticosteroids), delays total knee arthroplasty and reduces absences from work, lowering costs to patients and health care resources, in agreement with out results. Finally, the authors suggested that single injection may represent an interesting solution for low-income patients [56].

It is crucial to keep in mind that patients without intra-articular inflammation might respond better to HA treatment. Euppayo et al. [57], analyzing clinical and preclinical studies (13 papers), showed that intra-articular anti-inflammatory treatment with corticosteroids or NSAIDs plus HA was generally superior to HA alone in the management of OA (*p* < 0.001). However, the combination treatment may impair the expression of catabolic genes like aggrecan.

Exercise improves knee OA strength but not pain or impairment [58]. Despite contrasting evidence on this topic [59], Liles et al. [60] demonstrated that quadricep training did not increase the effectiveness of HA therapy.

Finally, our paper is the first, to our knowledge, that has also analyzed psychological status before and after the injection of HA in knee OA. Despite the good basal emotional well-being of our patients, we testify to a substantial time-related improvement in anxiety and depression, which are typically associated with chronic pain [61].

In our study, the clinical characteristics of enrolled patients excluded the presence of prior mental health interventions; moreover, clinical evaluation during the follow-up failed to account for the possibility that external life changes during the study period could have a role in mood states in these patients. In agreement with this observation, several authors documented that a decrease in pain can reduce depression, thus also improving the quality of life. Its crucial to keep in mind that improved psychological conditions improve patient–clinician relationships and increase treatment adherence [62]. Additionally, psychological well-being can support patients in maintaining an active lifestyle, which is crucial for osteoarthritis, particularly in order to prevent weight gain [63].

Investigating the mechanism behind the delays to total knee replacement due to cross-linked HA injections is not meaningless. A retrospective analysis of 182,022 knee OA patients who received total knee replacement [64] reported that 50,349 patients (27.7%) had at least one course of HA, while 131,673 patients (72.3%) did not receive it. The authors demonstrated that HA administration reduced the risk of total knee replacement (*p* < 0.0001). with a dose-dependent pattern.

HA’s mechanism of action appears to be linked to the stimulation of endogenous HA synthesis (viscoinduction) as well as biologic effects on cartilage and joint space, which also improve pain perception. Our study has several limitations: (1) The number of patients was relatively low and needs to be increased in a multicenter context in order to avoid the possible bias related to physician ability. (2) the study was conducted in a single center, and this could have limited the applicability of the results. (3) we did not consider gender-related factors (e.g., socio-economic and occupational status, job and religion) that could have played a role in the clinical efficacy of the drug formulation. (4) we enrolled only patients with a BMI < 29 to observe the effects of a single injection in a population with mild–moderate symptoms and low risk factors. (5) we did not have a control group, although the enrolled patients received systemic or local treatment before inclusion and therefore hey could be considered the control group at T0. We think that a larger randomized double-blind placebo-controlled multi-arm trial may help us to develop a better understanding of the effect of HA in a large group of patients. Finally, (6) we used subjective scales to measure clinical improvement, including NRS, SF-36 and KOOS. The use of magnetic resonance imaging (MRI) or vibroarthrography could be more accurate for evaluating the effects of HA in OA progression. Nevertheless, in our country, performing these exams before and after treatment is not appropriate given the associated economic burdens.

## 4. Materials and Methods

### 4.1. Study Design

This was a prospective longitudinal single-center clinical study on knee OA patients treated with cross-linked HA at the Pain Medicine Room of the Clinical Pharmacology and Pharmacovigilance Operative Unit of the “Renato Dulbecco” University Hospital of Catanzaro from January 2023 to December 2024. Patients were enrolled at baseline (T0) and then were monitored at 90 days (T1; 3 months), 180 days (T2; 6 months), and 360 days (T3; 12 months). At the beginning of the study, all enrolled patients signed the informed consent form before admission to the study.

This study was conducted in accordance with guidelines of the 1964 Declaration of Helsinki and its later amendments, and with full respect for patient privacy, as per Italian law (Ethics Committee authorization: 120/2018; ClinicalTrials.gov Identifier: NCT06814769, February 2025).

### 4.2. Patients

In this study, patients were enrolled according to the following eligibility criteria:

(1) Patients of both sexes between 18 and 65 years old, with a Body Mass Index < 29 kg/m^2^;
(2) Patients with second- or third-degree OA, according to the Kellgren–Lawrence classification of a weight-bearing radiograph of the knee taken no longer than one year ago;
(3) Patients with VAS (Visual Analogue Scale) intensities higher than 5/10 who did not respond to systemic medication therapy;
(4) Patients who understood the study’s objectives (and those who adhered to the orthopedist’s instructions, returned for follow-up, and completed the evaluation questionnaires);
(5) Patients able to provide their written informed consent to participate in the study and to use their data anonymously for scientific purposes.

Patients under 18 years of age were excluded. Othe exclusion criteria were as follows:

(1) Presence of an active malignancy of any type or history of malignancy;
(2) Grade I, mild, or advanced, grade IV, arthritis according to the Kellgren–Lawrence criteria;
(3) Local or systemic infection.
(4) Uncooperative patients, those suffering from neurological disorders and were therefore unable to follow the orthopedist’s instructions or unable to provide informed consent for participation in the study, or those who did not provide written consent;
(5) Any cases not described in the inclusion criteria.

### 4.3. Experimental Protocol

After the enrollment (T0) and during the follow-ups (T1–T3), clinical and laboratory data were collected directly by the medical staff involved in the study, and Zung SAS, Zung SDS and SF-36 questionnaires were administered. The dedicated database evaluated and recorded any systemic or local side effects, in accordance with our previous studies [65,66,67]. Cross-linked HMW HA (Diart ONE 90 mg/3 mL; Diaco Biofarmaceutici, Trieste, Italy) was administered in a single injection at the beginning of the study (T0). Before the admission to this study, all the enrolled patients received systemic treatments with NSAIDs and/or local treatment with corticosteroids, without clinical benefits. Therefore, we considered the time before administration as the control group.

#### 4.3.1. Questionnaires

The 36-Item Short Form Health Survey (SF-36) consists of 36 questions and was used to assess quality of life in relation to pathology and the effectiveness of treatment. Quality of life is defined as the subjective perception of one’s own well-being within their socio-cultural context or as the satisfaction of desires and pleasures. Questions are summarized into two component summary scores, the Physical Component Summary (PCS) and the Mental Component Summary (MCS) scores, representing eight concepts of health: physical functioning (PF), bodily pain (BP), role limitations due to physical health problems (RP), role limitations due to personal or emotional problems (RE), general mental health (MH), social functioning (SF), energy/fatigue or vitality (VIT), and general health perceptions (GH). A higher score represents better health while a low score corresponds to a lower quality of life [68,69].

Zung’s Self-Rating Anxiety Scale (Zung SAS) is a 20-item question scale that rates the four common characteristics of anxiety, both psychological and somatic. Responses are given on a 4-point scale, and range from 1 (none, or a little of the time) to 4 (most, or all of the time). The items include both negative and positive experiences. The final score ranges from 20 to 80 points. Anxiety is classified as normal (score 0 to 44), moderate (score 45 to 59) or severe (score 60 to 80) [70].

Zung’s Self-Rating Depression Scale (Zung SDS) is a 20-items question scale that rates the four common characteristics of depression. The items include psychological and physiological symptoms: 10 express negative experiences and 10 express positive experiences. Responses are given on a 4-point scale ranging from 1 (none, or a little of the time) to 4 (most, or all the time). Total raw scores range from 20 to 80. Depression is classified as normal (score 20 to 49), mild (score 50 to 59), moderate (score 60 to 69) or severe (score 70 to 80) [70].

The Knee Injury and Osteoarthritis Outcome score (KOOS) is used to evaluate the actual and long-term functional status of patients’ knees. It evaluates five domains: (1) pain; (2) other symptoms such as swelling, restricted range of motion and mechanical symptoms; (3) disability on the level of daily activities; (4) disability on a level physically more demanding than activities of daily living; (5) mental and social aspects such as awareness and lifestyle changes. A Likert scale is used, and all items have five possible answer options scored from 0 (No Problems) to 4 (Extreme Problems), while each of the five scores is calculated as the sum of the items included. Scores are transformed into a 0–100 scale, with 0 representing extreme knee problems and 100 representing no knee problems [71,72].

#### 4.3.2. Clinical Tests

The Timed Up and Go Test is commonly used by physicians to evaluate the risk of falling in the elderly. The patient rises from a chair, walks three meters away, turns, returns to the chair, and then sits down again. The time in which patients carry out this action is measured. A lower duration is suggestive of good functional performance, whereas a score of ≥13.5 s is generally considered the cut-off for estimating fall risk. Nevertheless, reported thresholds vary from 10 to 33 s in the literature. The test has also been used in osteoarthritis patients [73,74]. A previous meta-analysis by Bohannon et al. [75] documented mean values (95% confidence intervals) of 8.1 (7.1–9.0) seconds for 60- to 69-year-olds, 9.2 (8.2–10.2) seconds for 70- to 79-year-olds, and 11.3 (10.0–12.7) seconds for 80- to 99-year-olds.

The Six-Minute Walking Test is used to determine the submaximal aerobic/functional walking capacity. Generally, physicians measure the distance that patients walk in 6 min (normally 400–700 m in young and 300–400 in elderly). The test is completely self-paced by the patient, who are permitted to rest during the test, but without stopping the timer. In our study, we also evaluated the Visual Analogue Scale (VAS) and time of pain onset during this test [76,77].

#### 4.3.3. Efficacy End-Points

The first efficacy end-point was the statistically significant difference (*p* < 0.05) in the Knee Injury and Osteoarthritis Outcome Score (KOOS) measured at the follow-ups (T1–T3) compared with that at T0.

The secondary end-points were as follows:

- The statistically significant difference (*p* < 0.05) in the VAS between T2 and T0 and between T3 and T2;
- The statistically significant difference (*p* < 0.05) in functional mobility and walking ability (Six-minute Walking Test);
- The general differences between T2 and T0 and between T3 and T2;
- The statistically significant difference (*p* < 0.05), in general, health assessments (SF-36) between T2 and T0 and between T3 and T2.
- The statistically significant differences (*p* < 0.05) in mood disorders (Zung SAS and Zung SDS) between T2 and T0 and between T3 and T2.

#### 4.3.4. Safety End-Points

During the study, we recorded the development of adverse drug reactions related to the intra-articular injection of HA. We recorded the frequency, predictability, duration, severity, seriousness, course, and consequences of adverse drug reactions, and the ADRs leading to the withdrawal of subjects from the study.

### 4.4. Statistical Analysis

Gaussian continuous variables were described by means and standard deviations. Medians and interquartile range were used in cases of skewness. Counts and percentages were used for categorical variables. The normal distribution of continuous variables was verified by the Shapiro–Wilk test. A *t*-test was used to compare normally distributed continuous variables between males and females. A non-parametric Friedman test followed by post hoc analysis using Wilcoxon’s signed-rank test was also applied. Data are expressed as means ± standard deviations. For all comparisons, differences were considered significant for *p* < 0.05. Statistical analysis was performed using SPSS 22.0 (International Business Machines Corporation, Armonk, NY, USA) and JASP accessed on 10 June 2024 and 20 October 2024.

## 5. Conclusions

According to our research, intra-articular single injection of cross-linked hyaluronic acid therapy is a legitimate, secure, and successful conservative therapeutic choice for OA. This therapy is well tolerated and helps patients improve their mobility and pain management. Adverse effects associated with repeated injections are less likely when a single injection is used. Patients with symptomatic knee OA between the ages of 18 and 65, who may choose an alternate treatment option and who are not candidates for partial or total knee replacement, can benefit from a single injection of cross-linked high-molecular-weight HA.

## Figures and Tables

**Table 1:** Demographic characteristics of the enrolled patients. Data are expressed as means ± standard deviations for continuous variables and numbers (percentages) for categorical variables.

	Cross-Linked High-Molecular-Weight Hyaluronic Acid	
	Male	Female
Number, (%)	36 (72.0)	14 (28.0)
Age	49.44 ± 11.01	49.46 ± 12.63
Body max index (kg/m^2^)	25.60 ± 2.02	24.55 ± 2.91
Mean symptom duration (years)	4.06 ± 3.21	5.21 ± 4.32
Osteoarthritis; Kellgren–Lawrence classification	n (%)	n (%)
Stage I	5 (13.9)	2 (14.3)
Stage II	26 (72.2)	10 (71.4)
Stage II/III	2 (5.6)	0
Stage III	3 (8.3)	2 (14.3)

**Table 2:** Clinical- and functional-scale changes at T1 vs. T0 in patients treated with cross-linked high-molecular-weight hyaluronic acid. Data are expressed as medians (interquartile range). KOOS: Knee Injury and Osteoarthritis Outcome Score; VAS: Visual Analogue scale. ** *p* < 0.01.

Score	T0	T1	*p*
KOOS	44.90 ± 14.10	78.71 ± 12.68 **	0.000
VAS	6.90 ± 1.23	1.70 ± 1.37 **	0.000
Six-minute walking test (meters)	443 ± 160	580 ± 161 **	0.00
Walking test—VAS	7.16 ± 1.12	1.51 ± 1.40 **	0.000
Timed Up and Go Test (sec)	7.0 ± 2.1	5.4 ± 2.3 **	0.000

**Table 3:** Clinical and functional data recorded at T2 (6 months) and T3 (12 months) after the admission (T0), in patients treated with cross-linked high-molecular-weight hyaluronic acid. Data are expressed as means ± standard deviation. KOOS: Knee Injury and Osteoarthritis Outcome Score; VAS: Visual Analogue Scale. Significant values are in bold.

Score	T0	T2	*p* T2 vs. T0	T3	*p* T3 vs. T0
KOOS	44.90 ± 14.10	78.37 ± 20.54	**0.000**	48.31 ± 15.42	0.251
VAS	6.90 ± 1.23	1.85 ± 1.28	**0.000**	6.58 ± 2.15	0.363
Six-minute walking test (meters)	443 ± 160	578 ± 125	**0.000**	481 ± 136	0.203
Walking test—VAS	7.16 ± 1.12	1.38 ± 1.18	**0.000**	6.07 ± 3.26	**0.027**
Timed Up and Go Test (sec)	7.0 ± 2.1	5.45 ± 2.34	**0.000**	6.23 ± 1.46	**0.035**

**Table 4:** Outcomes of the 36-Item Short Form Health Survey (SF) recorded for the enrolled patients treated with cross-linked high-molecular-weight hyaluronic acid 3 months (T1) after admission (T0). Data are expressed as mean ± standard deviation. * *p* < 0.05; ** *p* < 0.01.

SF-36			
	T0	T1	*p*
Physical functioning	49.95 ± 9.35	62.28 ± 8.42 **	0.000
Role limitations due to physical health	59.40 ± 13.21	68.60 ± 10.96 **	0.000
Role limitations due to emotional problems	48.20 ± 5.84	61.56 ± 5.85 **	0.000
Energy/fatigue	56.00 ± 6.55	66.90 ± 7.68 **	0.000
Emotional well-being	59.50 ± 7.29	66.24 ± 13.75 **	0.002
Social functioning	57.70 ± 6.73	69.30 ± 6.73 **	0.000
Pain	47.70 ± 9.17	61.80 ± 9.80 **	0.000
General health	50.90 ± 11.57	56.40 ± 10.68 *	0.015
Health change	51.90 ± 8.02	63.20 ± 8.07 **	0.000

**Table 5:** Outcomes of the 36-Item Short Form Health Survey 36 (SF-36) recorded for the enrolled patients treated with cross-linked high-molecular-weight hyaluronic acid 6 months (T2) after admission (T0). Data are expressed as mean ± standard deviation. * *p* < 0.05; ** *p* < 0.01.

SF-36			
	T0	T2	*p*
Physical functioning	49.95 ± 9.35	60.03 ± 11.24 **	0.000
Role limitations due to physical health	59.40 ± 13.21	65.40 ± 7.51 **	0.006
Role limitations due to emotional problems	48.20 ± 5.84	61.15 ± 10.37 **	0.000
Energy/fatigue	56.00 ± 6.55	69.82 ± 15.44 **	0.000
Emotional well-being	59.50 ± 7.29	64.10 ± 12.56 *	0.027
Social functioning	57.70 ± 6.73	70.34 ± 13.45 **	0.000
Pain	47.70 ± 9.17	65.34 ± 10.28 **	0.000
General health	50.90 ± 11.57	55.32 ± 11.46	0.057
Health change	51.90 ± 8.02	62.38 ± 13.47 **	0.000

**Table 6:** Outcomes of the 36-Item Short Form Health Survey 36 recorded for the enrolled patients treated with cross-linked high-molecular-weight hyaluronic acid 12 months (T3) after admission (T0). Data are expressed as mean ± standard deviation. * *p* < 0.05; ** *p* < 0.01.

F-36			
	T0	T3	*p*
Physical functioning	49.95 ± 9.35	53.34 ± 9.53	0.075
Role limitations due to physical health	59.40 ± 13.21	62.18 ± 12.36	0.279
Role limitations due to emotional problems	48.20 ± 5.84	62.34 ± 11.47 **	0000
Energy/fatigue	56.00 ± 6.55	60.67 ± 14.52 *	0.040
Emotional well-being	59.50 ± 7.29	61.73 ± 9.24	0.183
Social functioning	57.70 ± 6.73	67.33 ± 12.85 **	0000
Pain	47.70 ± 9.17	51.74 ± 10.37 *	0.042
General health	50.90 ± 11.57	55.34 ± 12.48	0.068
Health change	51.90 ± 8.02	57.56 ± 15.47 *	0.023

**Table 7:** Outcomes of Zung depression and anxiety scales recorded for the enrolled patients treated with cross-linked high-molecular-weight hyaluronic acid 3 months (T1), 6 months (T2), and 12 months (T3) after admission (T0). Data are expressed as mean ± standard deviation. ** *p* < 0.01.

	**T0**	**T1**	***p***
Depression	61.30 ± 9.85	44.30 ± 8.31 **	0.000
Anxiety	56.60 ± 8.53	35.90 ± 9.51 **	0.000
	T0	T2	*p*
Depression	61.30 ± 9.85	46.50 ± 13.82 **	0.000
Anxiety	56.60 ± 8.53	32.35 ± 12.72 **	0.000
	T0	T3	*p*
Depression	61.30 ± 9.85	51.65 ± 11.28 **	0.000
Anxiety	56.60 ± 8.53	37.44 ± 9.51 **	0.000

## Data Availability

The original contributions presented in this study are included in the article. Further inquiries can be directed to the corresponding author.
