# Correction: Rania et al. Efficacy and Safety of Intra-Articular Therapy with Cross-Linked Hyaluronic Acid in Patients with Knee Osteoarthritis. *Pharmaceuticals* 2025, *18*, 302

**DOI:** 10.3390/ph18081131

**Published:** 2025-07-29

**Authors:** Vincenzo Rania, Gianmarco Marcianò, Cristina Vocca, Caterina Palleria, Luigi Bianco, Maria Cristina Caroleo, Luca Gallelli

**Affiliations:** 1 Operative Unit of Pharmacology and Pharmacovigilance, “Renato Dulbecco” University Hospital, 88100 Catanzaro, Italy; raniavincenzo1@gmail.com (V.R.); gianmarco.marciano3@gmail.com (G.M.); cristina_vocca@live.it (C.V.); palleria@unicz.it (C.P.); luigibianco1969@gmail.com (L.B.); mariacristina.caroleo@unicz.it (M.C.C.); 2 Research Center FAS@UMG, Department of Health Science, University Magna Graecia, 88100 Catanzaro, Italy

## Text Correction

In the original publication [1], there was an error in the Discussion section. The sentence originally stated:

“In our study, we excluded patients with a Body Mass Index lower than 29 kg/m^2^…”

This has been corrected to:

“In our study, we enclosed only patients with a Body Mass Index lower than 29 kg/m^2^…”

The authors state that the scientific conclusions are unaffected. This correction was approved by the Academic Editor. The original publication has been updated accordingly.
